# Phosphatidylinositol 4,5-Bisphosphate Mediates the Co-Distribution of Influenza A Hemagglutinin and Matrix Protein M1 at the Plasma Membrane

**DOI:** 10.3390/v14112509

**Published:** 2022-11-12

**Authors:** Prakash Raut, Bright Obeng, Hang Waters, Joshua Zimmerberg, Julie A. Gosse, Samuel T. Hess

**Affiliations:** 1 Department of Physics and Astronomy, University of Maine, Orono, ME 04469-5709, USA; 2 Department of Molecular and Biomedical Sciences, University of Maine, Orono, ME 04469-5735, USA; 3 Eunice Kennedy Shriver National Institute of Child Health and Human Development, National Institutes of Health, Bethesda, MD 20892-1855, USA

**Keywords:** influenza A, super-resolution fluorescence microscopy, host–virus interactions, matrix protein M1, phosphoinositides, PIP2, hemagglutinin, virus assembly, cetylpyridinium chloride, FPALM

## Abstract

The fully assembled influenza A virus (IAV) has on its surface the highest density of a single membrane protein found in nature—the glycoprotein hemagglutinin (HA) that mediates viral binding, entry, and assembly. HA clusters at the plasma membrane of infected cells, and the HA density (number of molecules per unit area) of these clusters correlates with the infectivity of the virus. Dense HA clusters are considered to mark the assembly site and ultimately lead to the budding of infectious IAV. The mechanism of spontaneous HA clustering, which occurs with or without other viral components, has not been elucidated. Using super-resolution fluorescence photoactivation localization microscopy (FPALM), we have previously shown that these HA clusters are interdependent on phosphatidylinositol 4,5-biphosphate (PIP2). Here, we show that the IAV matrix protein M1 co-clusters with PIP2, visualized using the pleckstrin homology domain. We find that cetylpyridinium chloride (CPC), which is a positively charged quaternary ammonium compound known for its antibacterial and antiviral properties at millimolar concentrations, disrupts M1 clustering and M1-PIP2 co-clustering at micromolar concentrations well below the critical micelle concentration (CMC). CPC also disrupts the co-clustering of M1 with HA at the plasma membrane, suggesting the role of host cell PIP2 clusters as scaffolds for gathering and concentrating M1 and HA to achieve their unusually high cluster densities in the IAV envelope.

## 1. Introduction

Influenza A virus (IAV) causes significant morbidity and mortality in human populations. IAV is an enveloped, negative-sense RNA virus in the *orthomyxoviridae* family [1]. The membrane contains viral glycoproteins, hemagglutinin (HA), neuraminidase (NA), and the matrix ion channel (M2) [2,3]. Adjacent to the inner leaflet of the viral membrane, matrix protein 1 (M1) forms a layer which stabilizes the virus particle through interactions with other viral components and the bilayer itself [4]. Finally, the viral core consists of nucleoprotein (NP), three polymerases (PB1, PB2, PA), eight RNA segments, and three nuclear export proteins (NEPs) [1]. The interaction of viral proteins with host cell membranes begins with viral binding to the host cell and continues through the viral life cycle until new progeny virions bud and are released [5,6,7,8]. Because the interruption of viral assembly halts viral disease, a better understanding of the interaction of viral components with host cell membranes is crucial to the development of new antiviral therapies.

M1 can bind to lipid bilayers through electrostatic interaction [9,10,11,12,13] and can form virus-like particles (VLPs) in the absence of other viral components [14,15], demonstrating its ability to interact with lipids, including cholesterol [16], in the plasma membrane (PM). Upon such binding, M1 multimerizes [17]. This formation of M1 multimers or clusters is suspected to play a crucial role in the viral life cycle, specifically in control of membrane curvature, viral morphology, and budding [3,18,19,20]. However, the role of HA in the clustering of M1 during interaction with the PM requires further elucidation, with some work showing little M1 clustering and minimal co-clustering of HA and M1 in infected cells [21], while other reports show the enhanced membrane association of M1 in the presence of HA [19] and evidence for an acylation- and curvature-dependent HA-M1 interaction [18]. Previous studies show that in model membranes and cell models of infection, M1 can bind to lipid bilayers containing PS [13,17,22], and that M1 and HA colocalize to some extent in co-transfected cells and to a lesser degree in infected cells [21]. However, there is less information about M1 binding to other lipids. While it is known that M1 can bind to lipid bilayers through multiple residues [9,16,23], that M1 is twice as likely to be adsorbed on phospholipidic surfaces than on neutral surfaces [24], and that M1 can preferentially deform giant unilamellar vesicles containing negatively charged lipids [25], whether M1 can interact specifically with phosphoinoisitides has not been extensively investigated. Our recent finding that HA and phosphatidylinositol 4,5-bisphosphate (PIP2) can interact to modulate HA clustering in the PM [5] raised the possibility that HA interactions with M1 [26,27] could be mediated by PIP2.

PIP2 is a minor component of the PM in mammalian cells (~1% of total lipid) but is the most abundant polyphosphoinositide [24,25] and plays an outsized role in cell function [28]. PIP2 mediates many intracellular processes, such as endo- and exocytosis [29,30], actin cytoskeleton regulation [31], cytoskeleton PM adhesion [32], and many others [28]. PIP2 clusters at the PM [6,33,34] and these clusters are known to interact with various cytoskeletal proteins such as actin and actin-binding proteins [35,36,37]. Proteins that have been purified from IAV are also known to interact with PIP2 [38], and IAV exploits several PIP2-dependent pathways [39,40]. Because other viruses, such as HIV and Ebola, use PIP2 to mediate the membrane binding of their capsid proteins, and because IAV HA and NP [6,41] have known interactions with PIP2, it is reasonable to investigate whether PIP2 and M1 might also interact.

We recently showed that the quaternary ammonium compound cetylpyridinium chloride (CPC) at non-cytotoxic concentrations modulates the membrane association and clustering of PIP2-binding proteins such as myristoylated alanine-rich C-kinase substrate (MARCKS) and phospholipase C (PLC)-δ through its pleckstrin homology (PH)-domain [42]. CPC contains a positively charged head group and a hydrophobic tail, allowing it to efficiently associate with membrane bilayers and micelles [43]. The detergent action of CPC is observed when the concentration of CPC is above its critical micelle concentration (CMC) in water [44], which has been quantified by various methods in different buffers and temperatures to be in the range ~600–900 µM [45,46,47,48].

Although CPC has been previously shown to possess antibacterial and antiviral properties through micelle formation at millimolar concentrations [49,50,51], there is much less information on how low micromolar concentrations of CPC could affect interactions between IAV components. Our recent CPC study showed that HA and PH (PIP2) co-clustering were modulated by CPC and suggested a mechanism for antiviral properties of CPC at non-cytotoxic concentrations [42]. In this study, we build on these findings and test the hypothesis that CPC modulates the assembly of HA and M1. Using the localization-based super-resolution microscopy technique of fluorescence photoactivation localization microscopy (FPALM) [52] with total internal reflection fluorescence (TIRF) excitation, we also shed light on the effect of M1 on the PM distribution of PIP2 and the effect of HA in the clustering of M1 adjacent to the PM.

## 2. Materials and Methods

### 2.1. Cell Culture, Transfection, and Fixation

NIH3T3 mouse fibroblast cells (ATCC, CRL-1658) were cultured in growth media (DMEM with glucose and L-glutamine, Lonza, 12-604F) with 10% calf bovine serum (30-2030, ATCC, Manassas, VA, USA) and antibiotics (penicillin streptomycin, 100 µg/mL) at 37 °C and 5% CO_2_ until they were 70–90% confluent. Cells were passed using 0.05% trypsin, were seeded at the concentration of 35,000 cells/mL in the Petri dish (P35G-1.5-20-C, MatTek, Ashland, MA, USA), and were grown in media (DMEM with glucose without phenol red 12-917F, Lonza Bioscience, Lexington, MA, USA) for 20–24 h in an incubator maintained at 37 °C and 5% CO_2_. After this time, cells were transfected using Lipofectamine 3000 (L3000008, Invitrogen/Thermo Fisher Scientific, Waltham, MA, USA) with 2 µg of total DNA (1 µg of either species in the case of two-color imaging). Cells were left in the same (previous) growth media overnight in the incubator and maintained at the above-mentioned conditions. After 20–24 h, cells were treated with control (0 µm CPC + Tyrode’s- BSA vehicle), 5 µM CPC, or 10 µM CPC for 1 h in the incubator maintained at 37 °C and 5% CO_2_. After an hour, Petri dishes were washed twice with phosphate-buffered saline (PBS) (D8537, Sigma-Aldrich, St. Louis, MO, USA) and fixed with 4% paraformaldehyde (PFA) (J61899AK, Alfa Aesar, Haverill, MA, USA) for approximately 15 min at room temperature and again washed twice with the PBS.

### 2.2. CPC Solution Preparation

Cetylpyridinium chloride (CPC; 99% purity, CAS no. 123–03-5, VWR/Avantor, Radnor, PA, USA) was prepared in aqueous Tyrode’s buffer, as described previously (Raut et al., 2022), without the use of organic solvent. CPC concentrations were checked via UV absorbance spectroscopy.

### 2.3. Two-Color FPALM Imaging

Two lasers of wavelengths 405 nm (Crystal Laser, 5 mW) and 558 nm (CrystaLaser, Reno, NV, USA, 100 mW) were aligned and focused into the back aperture plane of an oil immersion objective (60 × 1.45 NA, Olympus, Tokyo, Japan) by a lens (Thorlabs, Newton, NJ, USA) of focal length 350 mm. The 405 nm laser is used for the activation of fluorophores and the 558 nm laser is used for readout. The laser power entering the objective was recorded as ~75 µW and ~13 mW for the activation and read-out laser, respectively, using a power meter (Thorlabs, Newton, NJ, USA). Both the lasers were roughly circularly polarized before entering the objective for better activation and readout of the sample, first by passing the activation laser through a half-wave plate (10RP42-1, Newport, Irvine, CA, USA) and a linear polarizer (5511, Newport, Irvine, CA, USA), and then by passing both lasers through a quarter-wave plate (10RP54-1B, Newport, Irvine, CA, USA). The quad-band dichroic (Di01 R405/488/561/635-25x36, Semrock/IDEX, West Henrietta, NY, USA) inside the microscope reflects the laser into the objective, resulting in the widefield illumination of the sample. The incoming beam was then translated relative to the optical axis before entering the objective to result in an increased angle of incidence (measured relative to the optical axis) for the illumination; for sufficiently high angles of incidence, total internal reflection occurred at the glass–sample interface, allowing total internal reflection fluorescence imaging. Fluorescence from the sample was collected by the objective, then passed through the same dichroic through a notch filter (NF03-561E-25, Semrock/IDEX, West Henrietta, NY, USA), and then into the tube lens within the microscope. After (below) the tube lens, fluorescence was reflected by a glass beam splitter cube inside the microscope, resulting in a horizontal path outside of the microscope. Fluorescence then passed through an aperture and two achromatic lenses of focal lengths +20 mm and +40 mm, respectively, which served as a telescope with 2× magnification. Fluorescence emerging from the telescope reached a second dichroic (FF580-FDi02-t3, Semrock/IDEX, West Henrietta, NY, USA) within the multicolor module, which was aligned at 45° to reflect the fluorescence of wavelengths (λ) < 595 nm and transmit fluorescence with λ > 595 nm, thus creating two spectral channels for multi-color FPALM imaging [53]. Transmitted and reflected fluorescence pass through an ET630/92 filter (FF01-630/69-25, Semrock/IDEX, West Henrietta, NY, USA) for the “red” channel, or a 585/40 filter (FF01-585/40-25, Semrock/IDEX, West Henrietta, NY, USA) for the “green” channel, before reaching the camera (iXon+ DU897DCS-BV, Andor Scientific, Dublin, Ireland). At least ten thousand frames were recorded at ~32 Hz with an EM gain of 200. To maintain optimal molecular densities and reduce bleed-through, fluorescence from the M1-PAmKate molecules was recorded for the first five thousand frames, and then both species were imaged for the final five thousand frames.

### 2.4. Data Analysis

Raw images containing molecular images were analyzed using a custom-built Matlab code. After background subtraction [54], pixel values above a set threshold were sorted, and then areas of 7 × 7 pixels (“grabs”) containing at least one above-threshold pixel were selected, centered at the highest pixel value of the selected area. Each grab was then localized by fitting a 2D Gaussian function [52]. The total background-subtracted fluorescence in each grab in the red (F_R_) and green (F_G_) channels was used to determine the value alpha: α = F_R_/(F_R_ + F_G_) to allow different colors of molecular species to be separated [53]. After this, each localization was required to pass a minimum fit quality threshold to be included within the final dataset, which was then drift- and bleed-through-corrected [55].

### 2.5. Manders’ Colocalization Coefficient

Manders’ Colocalization Coefficient (MCC) for the green and red channels was computed as previously published [56]. Briefly, the localizations were separated into the two molecular species (“channels”) being imaged, according to their α values. After the separation of the species, data were binned separately in boxes of dimension 80 nm × 80 nm. A mask was drawn around each cell and all the bins inside the mask were included for the calculation. Next, all the pixels for one species (channel) were identified that had some (non-zero) pixel value of the second species (or that colocalized with the second species). Then, the pixel values (intensities) of the identified pixels were summed over, and this sum was divided by the sum of all the pixel values of the same species (channel). The resulting value was the MCC for one channel and was averaged over all the cells; similarly, MCC was computed for the second channel as well.

### 2.6. Cluster Identification

For cluster identification, we used the single linkage cluster analysis (SLCA) algorithm published previously [5,6,57,58,59,60]. In this method, each localization was assigned to either of the two channels according to their α values [6,53]. After this, the algorithm analyzes all localizations of the same channel using a cutoff distance d_max_. All the localizations within d_max_ or any other localization will be considered as in the same cluster. To threshold a minimum cluster size, the total localizations within a cluster were required to equal or exceed a certain value, min_pts. Here, the maximum intermolecular distance d_max_ = 50 nm and the minimum threshold of min_pts = 25 localizations within a cluster were used.

### 2.7. Co-Clustering Analysis: Mean Pixel Sum

Co-clustering analysis was performed by the Mean Pixel Sum method published previously [55]. Briefly, the localizations for each species were separated according to their α values, bleed-through-corrected, and then binned within a grid with pixels of dimension 80 nm × 80 nm. Next, a mask was drawn around the cell, and only the bins inside the mask were considered for further analysis. Co-clustering was quantified by calculating the mean molecule number per grid pixel for HA within M1 clusters (N_HA-M1_) and M1 within HA clusters (N_M1-HA_). Bins that had five or more localizations of one species were considered as a cluster of that species. Next, bins which had a cluster of the first species and at least some of the second species at the same position, and thus co-clustering, were included in the sum of localizations within that cluster, then divided by the number of grid pixels in the cluster. The number of localizations per grid pixel was then averaged over all the cells. A similar process was repeated for the second species.

### 2.8. Statistical Analyses

Tests of statistical significance of the differences between groups of data were performed in Graph Pad Prism 8.3.1. Data were copied to Graph Pad Prism directly from the Matlab files. Statistical significance was typically determined by one-way ANOVA followed by a Dunnett’s post hoc test for data comparing three or more groups, or using a Mann–Whitney test for data comparing only two groups.

### 2.9. Preparation of PAmKate-M1 Expression Vector

The M1-PAmKate vector was constructed such that PAmKate was in frame and at the C terminal of the Matrix 1 protein (A/Hong Kong/8/68). The over-expression vector pCAGGS M1 pH GFP was digested with Sac I and Nhe I (NEB, Ipswich, MA, USA). Digested products were loaded on the DNA gel and vectors containing M1 (without pH GFP) were purified using a Qiaquick PCR and gel clean-up kit (Qiagen, Germantown, MD, USA). PAmKate (Addgene) was amplified by PCR using mKate SacI F (forward oligo with SacI restriction site incorporated) and mKate NheI R (reverse oligo with NheI restriction side incorporated). The PCR products were digested with SacI and NheI and then purified with the Qiagen Kit. Vectors containing M1 and PAmKate PCR products were ligated and then transfected to *E. coli*, which grew on LB with an antibiotic to select for the ligated DNA containing both M1 and PAmKate. The final DNAs were purified from *E.coli* cultures and sequenced (Psomagen, Rockville, MD, USA) to confirm the correct DNA sequence. Correct DNAs of M1 PAmKate were purified on a larger scale using the Endo Free Plasmid Maxi Kit (Qiagen, Germantown, MD, USA) for further transfection into mammalian cell cultures.

## 3. Results

### 3.1. PIP2 and M1 Colocalization Are Disrupted by CPC

**Figure 1:**
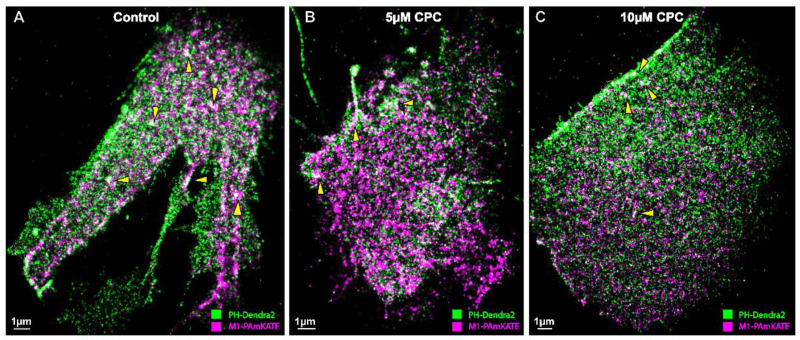
Super-resolution image showing the colocalization of PIP2 and M1. NIH3T3 cells expressing PH-Dendra2 (green) and M1-PAmKate (magenta) were exposed to control media (0 µM CPC+ Tyrode’s-BSA vehicle), 5 µM CPC, or 10 µM CPC, and were incubated at 37 °C for an hour. Cells were chemically fixed after an hour using 4% PFA. Two-color FPALM imaging of the PM of the cells was carried out in TIRF illumination. Cells treated with (**A**) control show a higher degree of colocalization (white) relative to the (**B**) 5 µM CPC and (**C**) 10 µM CPC treatments. The areas of co-clustering (white) decrease with the CPC treatment. Examples of colocalization (white) are shown by the yellow triangles. Scale bar = 1 μm.

**Figure 2:**
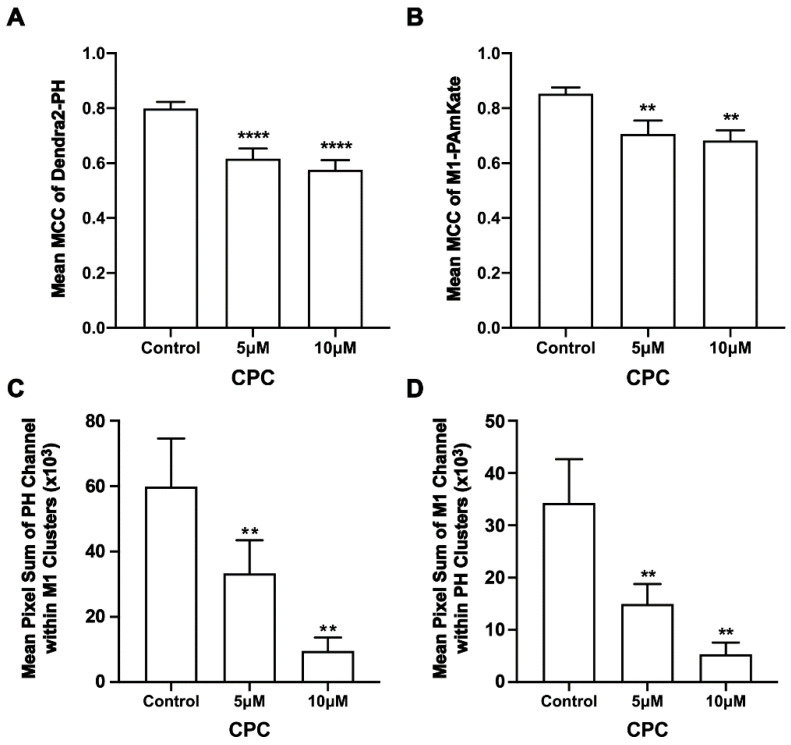
CPC reduces the colocalization and co-clustering of PIP2 and M1 in fixed NIH3T3 cells. NIH3T3 cells expressing PH-Dendra2 and M1-PAmKate were exposed to control media (0 µM CPC+ Tyrodes-BSA vehicle), 5 µM CPC, and 10 µM CPC and incubated at 37 °C for an hour. After an hour, cells were fixed using 4% PFA. Two-color FPALM imaging of PH-Dendra2 and M1-PAmKate was carried out with TIRF illumination. Localizations were assigned to one of the two channels according to their α values and were binned in pixels of dimensions 80 nm × 80 nm. (**A**,**B**) The Manders’ Colocalization Coefficient (MCC) was quantified and plotted as a function of CPC treatment. Both the MCC values—(**A**) PH-Dendra2 with PAmKate and (**B**) M1-PAmKate with PH-Dendra2—decreased significantly with CPC treatment. Values presented are mean ± SEM for a total of 33 cells for control, 31 cells for 5 µM, and 32 cells for the 10 µM CPC treatment from three independent experiments. Statistically significant results are represented by **** (*p* < 0.0001) and ** (*p* < 0.01) determined by ordinary one-way ANOVA. (**C**,**D**) Pixels containing clusters (defined as at least 5 localizations of a given species) were identified, and for each such pixel a sum of the number of localizations of M1 and PH was calculated, then averaged. This mean sum of localizations per pixel is defined as the Mean Pixel Sum (see methods) and was used as a measure of co-clustering of PH domain clusters with the M1 clusters. A similar analysis was performed on the pixel sum of M1 localizations co-clustering with PH domain clusters. (**C**) Mean Pixel Sum of PH domain co-clustering with M1 and (**D**) Mean Pixel Sum of M1 co-clustering with PH domain were plotted as functions of CPC treatment.

Given M1 binds to membranes primarily through electrostatic interactions [9,13], we tested the colocalization of M1 with PIP2 and if CPC affected any measured colocalization of M1 with PIP2. For this, two-color super-resolution microscopy with TIRF illumination was used to image the PM of fixed NIH3T3 cells expressing Dendra2-PH (PLC-δ) and M1-PAmKate. PIP2 colocalized with M1 at the PM (Figure 1). The colocalization and co-clustering were quantified using Manders’ Colocalization Coefficient (MCC) and Mean Pixel Sum, respectively (Figure 2). An MCC of 0.79 ± 0.02 was obtained for Dendra2-PH (PLC-δ) with M1-PAmKate, and an MCC of 0.85 ± 0.02 was obtained for M1-PAmKate with Dendra2-PH. Upon CPC treatment with concentrations of 5 µM and 10 µM, the MCC value for Dendra2-PH (PLC-δ) decreased significantly (0.62 ± 0.04 and 0.58 ± 0.04, respectively, *p* < 0.0001, using ordinary one-way ANOVA followed by followed Dunnett’s multiple comparison test against the control) (Figure 2). Similarly, the MCC value for M1-PAmKate also decreased significantly (*p* < 0.01, using ordinary one-way ANOVA followed by Dunnett’s multiple comparison test against the control) to 0.71 ± 0.05 and 0.68 ± 0.04 with 5 µM and 10 µM CPC treatment, respectively (Figure 2).

### 3.2. CPC Disrupts PIP2 Clusters in NIH3T3 Cells Expressing M1

**Figure 3:**
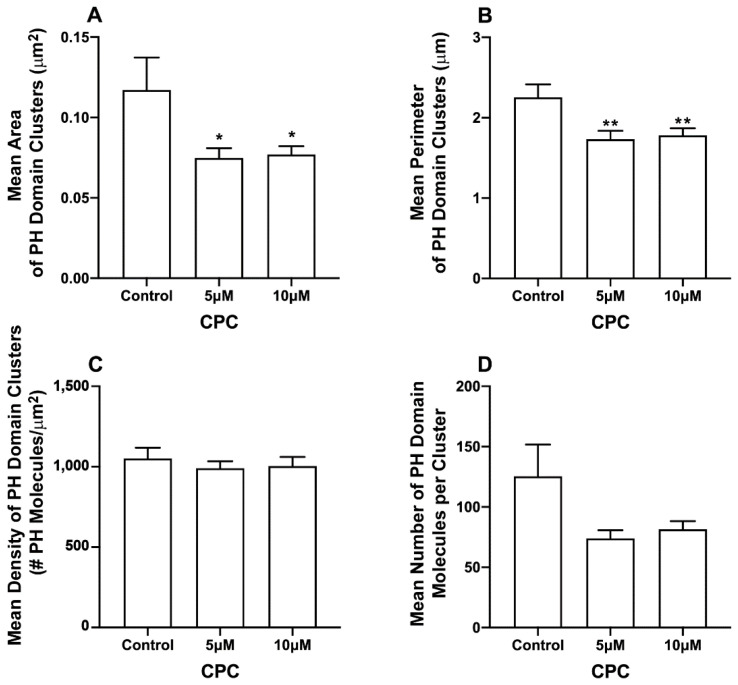
CPC modulates PIP2 clustering in fixed NIH3T3 cells expressing M1. NIH3T3 cells expressing PH-Dendra2 and M1-PAmKate were exposed to control media (0 µM CPC+ Tyrodes-BSA vehicle), 5 µM CPC, or 10 µM CPC and were incubated at 37 °C for 1 h. Cells were fixed using 4% PFA after the incubation, and then imaged by two-color FPALM with TIRF illumination. Cluster properties of the PH-Dendra2 clusters—(**A**) mean area, (**B**) mean perimeter, (**C**) mean density, and (**D**) mean number of localizations per cluster—were quantified using SLCA and plotted as a function of CPC treatment. Values presented are mean ± SEM for a total of 33 cells for control, 31 cells for 5 µM and 32 cells for the 10 µM CPC treatment, pooled from three independent experiments. Statistically significant results are represented by * *p* < 0.05 and ** *p* < 0.01 determined by ordinary one-way ANOVA.

**Figure 4:**
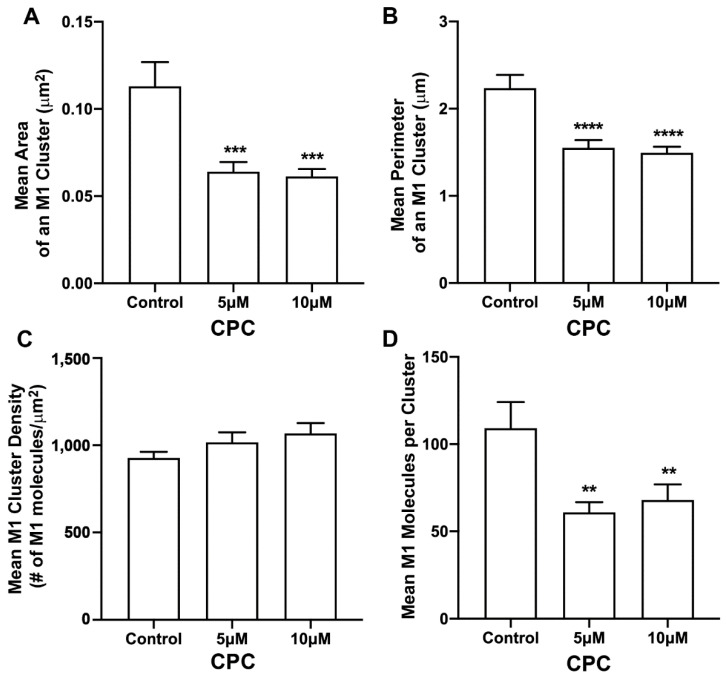
CPC modulates M1 clustering in fixed NIH3T3 cells. NIH3T3 cells expressing PH-Dendra2 and M1-PAmKate were exposed to control media (0µM CPC+ Tyrode’s-BSA vehicle), 5 µM CPC or 10 µM CPC, and were incubated at 37 °C for an hour and fixed at room temperature using 4% PFA. PMs of fixed cells expressing PH-Dendra2 and M1-PAmKate were imaged using FPALM in TIRF illumination. Cluster analysis was performed using SLCA. Cluster properties—(**A**) mean area of a M1 cluster, (**B**) mean perimeter of a M1 cluster, (**C**) mean density of a M1 cluster, and (**D**) mean number of M1 molecules forming a M1 cluster—were quantified and plotted as a function of CPC treatment. Values presented are mean ± SEM for a total of 33 cells for control, 31 cells for 5 µM, and 32 cells for the 10 µM CPC-treated cells, pooled from three independent experiments. Statistically significant results are represented by **** *p* < 0.0001, *** *p* < 0.001 and ** *p* < 0.01 determined by ordinary one-way ANOVA.

**Figure 5:**
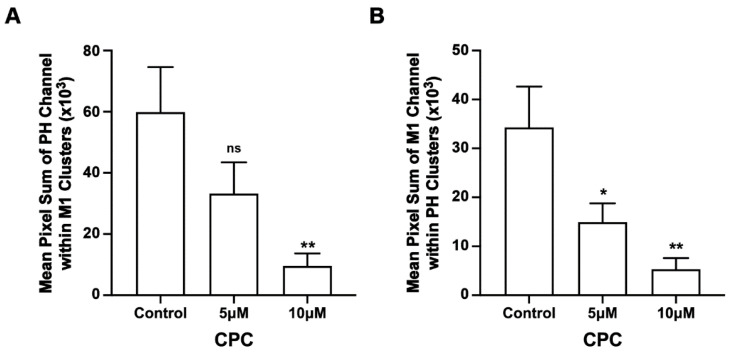
CPC reduces the co-clustering of PIP2 and M1. NIH3T3 cells expressing PH-Dendra2 and M1-PAmKate were exposed to control media (0 µM CPC+ Tyrode’s-BSA vehicle), 5 µM CPC, and 10 µM CPC, and were incubated at 37 °C for an hour. Cells were chemically fixed using 4% PFA at room temperature. Two-color FPALM was carried out with TIRF illumination to image the PMs (lower surface) of the cells. After the separation of their color according to the α value, localizations were binned in pixels of dimensions 80 nm × 80 nm. Pixels containing clusters (at least five localizations of one of the two species) were identified. The mean sum of localizations in these pixels was defined as the Mean Pixel Sum and was used as a measure of co-clustering PH domain localizations with M1 clusters. A similar analysis was performed on the pixel sum of M1 localizations co-clustering with PH domain clusters. (**A**) Mean Pixel Sum of PH domain co-clustering with M1 and (**B**) Mean Pixel Sum of M1 co-clustering with PH domain were plotted as functions of CPC treatment. Values presented are mean ± SEM for a total of 33 cells for control, 31 cells for 5 µM, and 32 cells for the 10 µM CPC-treated cells, pooled from three independent experiments. Statistically significant results compared to control are represented by * (*p* < 0.05) or ** (*p* < 0.01) determined by ordinary one-way ANOVA with Dunnett’s multiple comparison test.

The pleckstrin homology (PLC-δ) domain binds to PIP2 by specific electrostatic interactions and hydrogen bonds and can be used as a marker of unbound PIP2 in cellular membranes [6,61]. CPC disrupts the clustering of PIP2-binding proteins, and in some cases, their binding to the PM [42]; since HA interacts with PIP2, the above result led us to examine the effect of CPC on clusters of the PH domain [33,34] in the presence and absence of M1 expression. For this, two-color super-resolution microscopy was used to image the PM of fixed NIH3T3 cells expressing the PH domain protein tagged with Dendra2 and IAV matrix protein M1 tagged with PAmKate. Cluster and co-cluster analyses were performed using SLCA and Mean Pixel Sum (see methods) (Figure 3, Figure 4 and Figure 5). Upon the quantification of cluster properties, the mean area of a PH domain cluster (Figure 3) for the control was found to be 0.12 ± 0.02 µm^2^, which decreased to 0.075 ± 0.006 µm^2^ and 0.077 ± 0.005 µm^2^ with the CPC treatment of the concentration 5 µM and 10 µM, respectively. This decrement was significant (*p* < 0.05) by one-way ordinary ANOVA. When followed by the Dunnett’s multiple comparison test, only the 5 µM CPC treatment was significantly different (*p* < 0.05) from the control, while the 10 µM CPC treatment was barely insignificant (*p* = 0.056). Similarly, the mean perimeters of the PH domain clusters also decreased to 1.73 ± 0.10 µm and 1.78 ± 0.09 µm, respectively, for 5 μM and 10 μM CPC, when compared to the perimeter of the control (2.25 ± 0.16 µm). The mean perimeter result was significant (*p* < 0.01) when tested by ordinary one-way ANOVA followed by Dunnett’s multiple comparison test against the control. The mean density value of PH domain clusters decreased slightly from 1051 ± 67/µm^2^ (control) to 990 ± 43/µm^2^ and 1004 ± 57/µm^2^ for 5 µM and 10 µM CPC-treated cells, respectively. However, differences in the mean density of the PH domain cluster were insignificant by ordinary one-way ANOVA, followed by Dunnett’s multiple comparison test against the control. The mean number of PH molecules forming a PH domain cluster also decreased for 5 µM and 10 µM CPC treatment to 74 ± 7 and 82 ± 7, respectively, when compared to the control, where 125 ± 26 PH molecules were found forming a PH domain cluster, on average, but this was not significant by ordinary one-way ANOVA followed by Dunnett’s multiple comparison test against the control. Thus, CPC did affect the area and perimeter, but not density and molecule number, of the PH-labeled PIP2 clusters in cells expressing M1.

### 3.3. CPC Disrupts M1 Clusters in NIH3T3 Cells

Next, the effect of CPC on the distribution of M1 found with PH clusters was studied. For this, two-color FPALM was used to image the PM of NIH3T3 cells expressing M1-PAmKate and Dendra2-PH (PLC-δ) using TIRF illumination. M1 clusters were identified, and the cluster properties were quantified by SLCA (Figure 4). The mean cluster area of M1 clusters for the control was found to be 0.113 ± 0.014 µm^2^, which reduced to 0.064 ± 0.005 µm^2^ and 0.061 ± 0.004 µm^2^ for 5 µM and 10 µM CPC-treated cells, respectively. The mean area of M1 clusters significantly decreased (*p* < 0.001, ordinary one-way ANOVA followed by Dunnett’s test against the control) with CPC treatment. Similarly, the mean perimeter of an M1 cluster for the control was observed to be 2.24 ± 0.15 µm, which decreased significantly to 1.55 ± 0.09 µm (*p* < 0.0001, ordinary one-way ANOVA followed by Dunnett’s test against the control) and 1.49 ± 0.07 µm upon treatment with CPC concentrations of 5 µM and 10 µM, respectively. The mean density of the M1 clusters for the control was observed to be 930 ± 30/μm^2^, which slightly increased to 1020 ± 60/µm^2^ and 1070 ± 60/µm^2^ with the 5 µM and 10 µM CPC treatment, respectively. The mean value of the density of M1 clusters seemed to increase slightly with the CPC treatment, but no significant difference was observed by ordinary one-way ANOVA followed by Dunnett’s multiple comparison test against the control. On average, about 109 ± 15 molecules were found in a M1 cluster for the control, while only 61 ± 6 and 68 ± 9 M1 molecules were found in a M1 cluster in the cells treated with 5 µM and 10 µM CPC, respectively. This decrease was significant (*p* < 0.01) by ordinary one-way ANOVA followed by Dunnett’s multiple comparison test against the control. Thus, CPC modulates the PM distribution of M1.

### 3.4. CPC Disrupts the Co-Clustering of PIP2 and M1 in NIH3T3 Cells

We found that CPC modulated M1 clustering in the PM; to test if CPC specifically affected the nanoscale association of PH and M1, we performed two-color FPALM of PH and M1 ± CPC. The co-clustering of PH with M1 was reduced by approximately 44% and 84% with 5 µM and 10 µM CPC treatment, respectively (Figure 5). This decrement in the pixel sum (see methods, Figure 5 caption) with CPC treatment was significant (*p* < 0.01) by ordinary one-way ANOVA, but when followed by Dunnett’s multiple comparison test against the control, only the co-clustering result of 10 µM CPC treatment was significant (*p* < 0.01). Similarly, the co-clustering of M1 with PH was also reduced by approximately 56% and 85% with 5 µM and 10 µM CPC treatment, respectively, and was significant (*p* < 0.01) by ordinary one-way ANOVA. When followed by Dunnett’s multiple comparison test against the control, both the 5 µM and 10 µM CPC treatments were significantly different from control, with *p* < 0.05 and *p* < 0.01, respectively. Thus, CPC modulates whether M1 and the PIP2 marker PLCδ-PH are together within the same nanoscale regions.

### 3.5. M1 Enhances PIP2 Clustering

**Figure 6:**
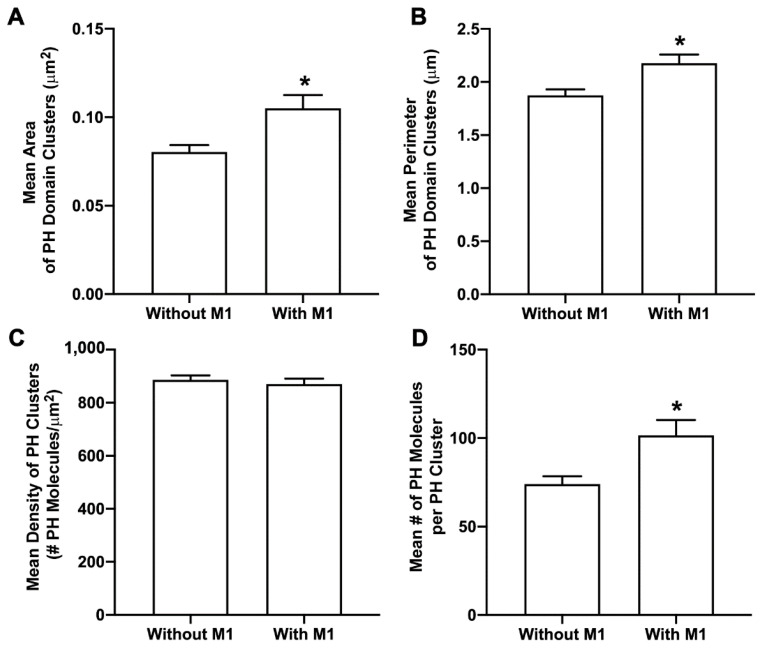
M1 enhances PIP2 clustering. Cells expressing Dendra2-PH with and without M1-PAmKate were fixed using 4% PFA and imaged by two-color FPALM with TIRF illumination. Cluster properties—(**A**) mean area of a PH domain cluster, (**B**) mean perimeter of a PH domain cluster, (**C**) mean density of a PH domain cluster, and (**D**) mean number of molecules forming a PH domain cluster—were quantified using SLCA and compared ±M1. Values presented are mean ± SEM for a total of 29 cells expressing PH domain only (without M1) and 36 cells expressing PH domain and M1, pooled from three independent experiments. Statistically significant results are represented by * when *p* < 0.05, as determined by an ordinary Mann–Whitney test.

To understand the effect of M1 on PIP2, NIH3T3 cells expressing Dendra2-PH(PLCδ) only and Dendra2-PH(PLCδ) with IAV M1-PAmKate were fixed using PFA (4%) at room temperature. Two-color FPALM was used to image the PM of fixed cells with TIRF illumination. Clusters of PH domains with and without M1 were quantified using SLCA (Figure 6). The mean cluster area of PH domain clusters significantly increased (*p* < 0.05 using a two-tailed Mann–Whitney test) from 0.080 ± 0.004 µm^2^ in the absence of M1 to 0.105 ± 0.008 µm^2^ when co-expressed with M1. Similarly, the perimeter of the PH domain clusters also significantly increased (*p* < 0.05 using a two-tailed Mann–Whitney test). The perimeter of the PH domain clusters without the expression of M1 was 1.87 ± 0.06 µm and the mean perimeter of a PH domain cluster with the expression of M1 was found to be 2.18 ± 0.08 µm. The mean density of a PH domain cluster without the expression of M1 was observed to be 886 ± 17/µm^2^, and with the expression of M1 was 870 ± 21/µm^2^. The number of molecules found in a PH domain cluster without the expression of M1 was 74 ± 4, which increased to 102 ± 9 with the co-expression of M1 (not significant by two-tailed Mann–Whitney test). However, the mean density of a PH domain cluster with and without M1, and the number of molecules in a cluster did increase significantly (*p* < 0.05 by two-tailed Mann–Whitney test). Thus, M1 was observed to modulate the PM distribution of PIP2 visualized by Dendra2-PH.

### 3.6. CPC Disrupts the Colocalization of HA and M1 Expressed Together in NIH3T3 Cells

**Figure 7:**
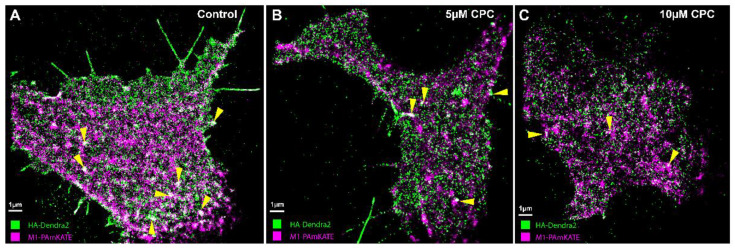
Colocalization of HA and M1 is CPC-dependent. NIH3T3 cells transfected with influenza A HA-Dendra2 and M1-PAmKate were treated with the control (0 µM CPC+ Tyrode’s-BSA vehicle), 5 µM CPC, or 10 µM CPC and incubated at 37 °C for an hour, then chemically fixed using PFA (4%) at room temperature. The lower PM of the cells was imaged with FPALM using TIRF illumination. HA-Dendra2 is rendered in green and M1-PAmKate in magenta. Yellow triangles point to the regions of colocalizations (white). Cells treated with the (**A**) control show a higher degree of colocalization compared to (**B**) 5 µM CPC and (**C**) 10 µM CPC-treated cells.

**Figure 8:**
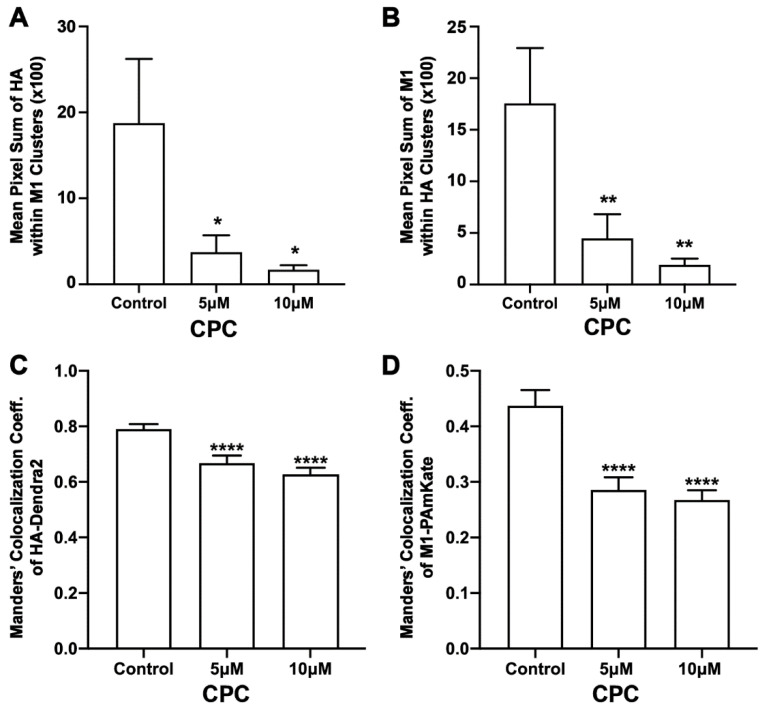
CPC reduces the co-clustering of HA and M1 in NIH3T3 cells. NIH3T3 cells transfected with influenza A HA-Dendra2 and M1-PAmKate were treated with the control (0 µM CPC+ Tyrode’s-BSA vehicle), 5 µM CPC, and 10 µM CPC and incubated at 37 °C for an hour and fixed with PFA (4%). Localizations from the two-color FPALM imaging of the PM of the fixed cells expressing HA-Dendra2 and M1-PAmKate were assigned to either channel according to their α value. Assigned localizations were binned in pixels of dimensions 80 nm × 80 nm and the Mean Pixel Sum was calculated (see methods) as a measure of the co-clustering of HA and M1. (**A**) Mean Pixel Sum of HA clusters containing M1 and (**B**) Mean Pixel Sum of M1 clusters containing HA vs. CPC treatment. (**C**,**D**) Manders’ Colocalization Coefficients (**C**) (MCCs) of HA-Dendra2 with PAmKate-M1 and (**D**) M1-PAmKate with Dendra2-HA reduced significantly with CPC treatment. Values presented are mean ± SEM for a total of 33 cells for control, 28 cells for 5 µM, and 30 cells for the 10 µM CPC-treated cells from three independent experiments. Statistically significant results are represented by * (*p* < 0.05), ** (*p* < 0.01), and **** (*p* < 0.0001) determined by ordinary one-way ANOVA.

Previous work has shown that HA and M1 interact [19,26,27]. If PIP2 plays a role in the HA-M1 interaction, we hypothesized that CPC would perturb this interaction. To test this hypothesis, two-color super-resolution microscopy was used to image HA-Dendra2 [6] and M1-PAmKate at the PM of transfected, fixed NIH3T3 cells using TIRF illumination. HA colocalized with M1 at the PM (Figure 7), so to test the effect of CPC on HA and M1, the MCC was quantified as a function of CPC treatment (Figure 8). A relatively high colocalization of HA with M1 was observed, compared to that of M1 with HA: a colocalization coefficient of 0.79 ± 0.02 was observed for HA with M1, while a correlation value of only 0.44 ± 0.03 was observed for M1 with HA. CPC treatment caused the MCC values to decrease significantly (*p* < 0.0001) by ordinary one-way ANOVA followed by Dunnett’s multiple comparison test against the control. The HA colocalization coefficient with M1 decreased by approximately 15% and 20% to 0.67 ± 0.03 and 0.63 ± 0.03 upon CPC treatment with 5 µM and 10 µM, respectively (Figure 8). Similarly, the M1 colocalization coefficient values decreased by approximately 34% and 39% to 0.29 ± 0.02 and 0.27 ± 0.02 upon treatment with CPC concentrations of 5 µM and 10 µM, respectively (Figure 8).

The co-clustering of HA and M1 was also quantified by the Mean Pixel Sum metric (see methods). The Mean Pixel Sum of the HA localizations within the M1 clusters (Figure 8) was reduced by approximately 80% and 91% with 5 µM and 10 µM CPC treatments, respectively, when compared to the control. This result was significant by ordinary one-way ANOVA. With the post-Dunnett’s test, 5 µM CPC treatment was not significant (*p* = 0.66) when compared against the control, but was significant with 10 µM CPC treatment (*p* < 0.05). The mean pixel sum of M1 clusters together with HA clusters was also observed to be reduced by about 75% and 89% upon treatment with 5 µM and 10 µM CPC, respectively, compared to the control. This decrease in the co-clustering of M1 with HA was significant (*p* < 0.01) by ordinary one-way ANOVA followed by post-Dunnett’s multiple comparison test against the control. Thus, CPC was found to modulate HA-M1 co-clustering at the nanoscale.

### 3.7. CPC Disrupts M1 Clusters in NIH3T3 Cells Expressed Together with HA

**Figure 9:**
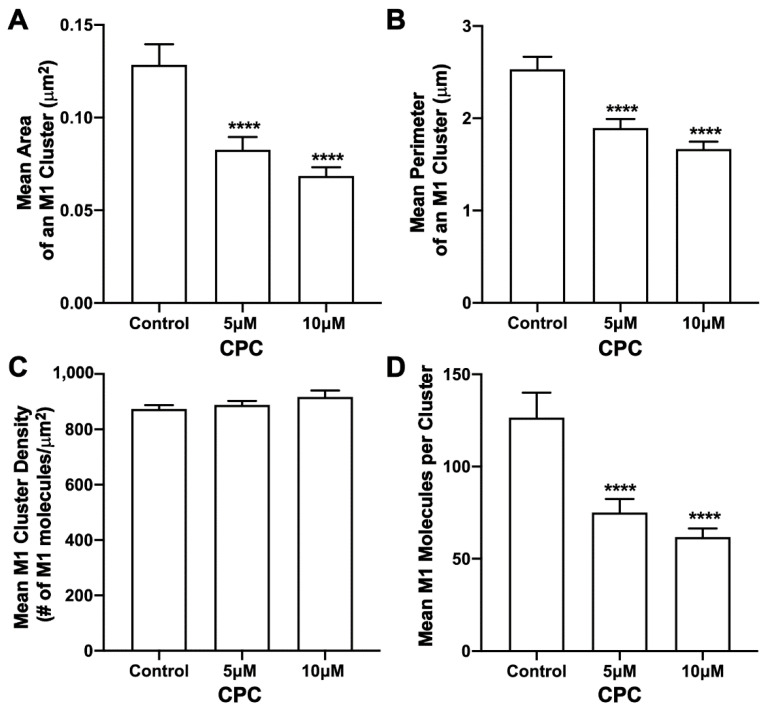
CPC modulates M1 clustering in NIH3T3 cells co-expressing HA and M1. NIH3T3 cells transfected with influenza A HA-Dendra2 and M1-PAmKate were treated with the control (0 µM CPC+ Tyrode’s-BSA vehicle), 5 µM CPC, or 10 µM CPC and incubated at 37 °C for an hour and chemically fixed with PFA (4%) at room temperature. Two-color FPALM imaging of the PM of the fixed NIH3T3 cells expressing HA-Dendra2 and M1-PAmKate was carried out under TIRF illumination. Localizations were assigned to one of the two-color channels according to their α values, then further processed through SLCA for cluster identification. Cluster properties—(**A**) mean area of an HA cluster, (**B**) mean perimeter of an HA cluster, (**C**) mean density of an HA cluster, and (**D**) mean number of molecules forming a cluster—were quantified and plotted as a function of CPC treatment. Values presented are mean ± SEM for a total of 34 cells for control, 29 cells for 5 µM, and 32 cells for the 10 µM CPC-treated cells, combined from three independent experiments. Statistically significant results are represented by **** (*p* < 0.0001) determined by ordinary one-way ANOVA.

The effect of CPC on M1 clusters was also studied when M1 was expressed together with HA as HA-Dendra2 and M1-PAmKate in NIH3T3 mouse fibroblast cells. Control (0 µM CPC), 5 µM, and 10 µM CPC conditions were used for an hour at 37 °C, followed by PFA fixation at room temperature, and the PM of fixed cells were imaged with FPALM with TIRF illumination. M1 clusters and HA were quantified using SLCA; upon quantification (Figure 9), M1 clusters had a mean area of 0.128 ± 0.011 µm^2^ for the control, and 0.083 ± 0.007 µm^2^ and 0.069 ± 0.005 µm^2^ for 5 µM and 10 µM CPC-treated cells, respectively. The decrement observed was approximately 35% and 46% with 5 µM and 10 µM CPC treatments, respectively, and was significant by ordinary one-way ANOVA followed by Dunnett’s multiple comparison test against the control. A similar result was obtained for the perimeter of an M1 cluster. The mean perimeter of an M1 cluster was found to be significantly (*p* < 0.0001) decreased from 2.53 ± 0.14 µm (control) by about 25% to 1.89 ± 0.10 µm for 5 µM, and by about 35% to 1.67 ± 0.08 µm for 10 µM CPC-treated cells when tested by ordinary one-way ANOVA followed by Dunnett’s multiple comparison test against the control. The mean density of M1 clusters was observed to increase slightly with CPC treatment, but no significant difference was observed by ordinary one-way ANOVA followed by Dunnett’s multiple comparison test against the control. The mean density of M1 clusters for the control, 5 µM CPC, and 10 μM CPC, were observed to be 873 ± 15/µm^2^, 888 ± 15/µm^2^, and 917 ± 23/µm^2^, respectively. While no significant difference was observed in the density, the mean number of M1 molecules forming a cluster was found to decrease significantly (*p* < 0.0001) by ordinary one-way ANOVA followed by Dunnett’s multiple comparison test against the control. On average, about 127 ± 14 molecules per cluster were found in the cells treated with the control media, which decreased by about 40% and 51% to 75 ± 7 M1 molecules and 62 ± 5 M1 molecules per cluster with the CPC treatment of concentrations 5 µM and 10 µM, respectively. Thus, CPC reduced the size of M1 clusters without affecting their average density.

### 3.8. HA Does Not Modulate M1 Association with the Membrane

**Figure 10:**
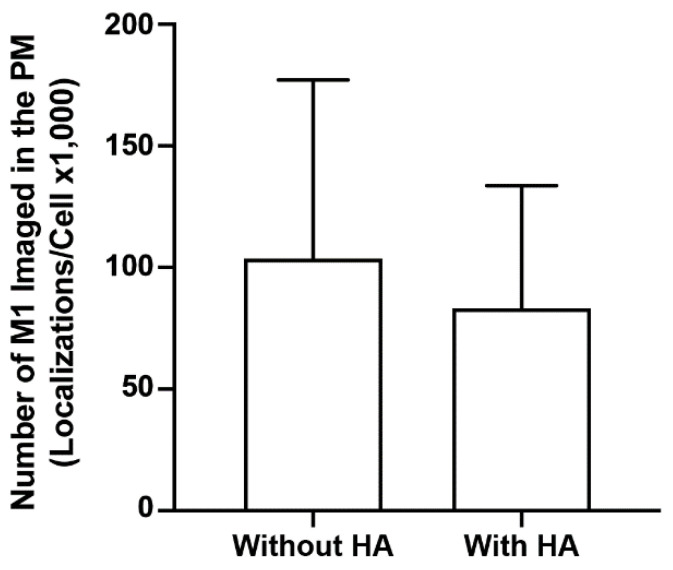
HA does not modulate M1 association with the plasma membrane. The mean numbers of M1 localizations in imaged regions of fixed NIH3T3 cells expressing PAmKate-M1 with or without HA imaged TIRF excitation were calculated and compared. Values presented are mean ± SD for a total of 34 cells without HA, and 33 cells with HA, from three independent experiments. No statistical significance was observed with the one-tailed Mann–Whitney test.

It is generally thought that the nature of the interaction between M1 and the lipid membrane is electrostatic. However, studies show conflicting results about the role of viral glycoproteins in the membrane association of M1. In one study, M1 association was affected by the viral glycoproteins HA and NA [26], while other studies showed that co-expression with the viral glycoproteins did not alter the membrane association of M1 significantly [10,62]. To explore this controversy, the number of localized M1 molecules using super-resolution FPALM with TIRF illumination was compared with and without HA expression. The number of M1 localizations per cell without HA (but expressed with the PH domain) was compared with the number of M1 localizations expressed together with HA (Figure 10). Although the mean number of localizations was slightly greater for the M1 expressed without HA, no significant difference was observed by either the one- or two-tailed Mann–Whitney test. Thus, HA appears not to directly modulate the number of M1 localizations within the PM under the conditions tested here.

### 3.9. HA Enhances M1 Clustering at the PM of NIH3T3 Cells

**Figure 11:**
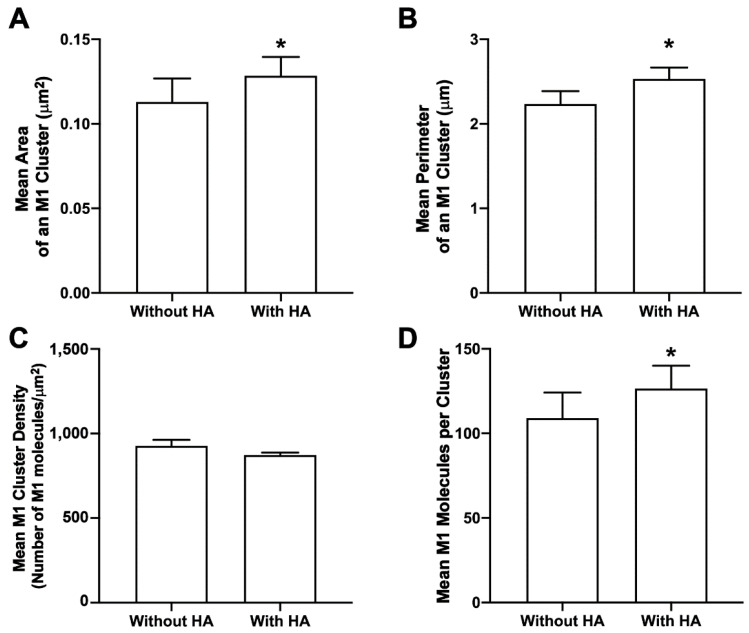
HA enhances M1 clustering in NIH3T3 cells. Cluster properties—(**A**) mean area of an M1 cluster (**B**), mean perimeter of an M1 cluster, (**C**) mean density of an M1 cluster, and (**D**) mean number of M1 molecules forming a cluster—were compared with and without HA. Values presented are mean ± SEM for a total of 33 cells without HA and 33 cells with HA from three independent experiments. Statistically significant results are represented by * (*p* < 0.05) determined by the one-tailed Mann–Whitney test.

M1 multimerizes after binding to the lipid bilayer [17]. This multimerization may have important implications in the IAV life cycle, as M1 can induce lipid deformation after multimerization [25] and can also form VLPs on its own by causing outward membrane protrusions [14]. The role of viral glycoprotein(s) in M1 clustering at the PM has not been fully explored. M1 cluster properties were compared for cells with and without HA expression (but expressed along with PH domain in both conditions). When expressed together with HA, there was a significant increase in mean M1 cluster area, perimeter, and the number of M1 molecules forming a cluster (one-tailed Mann–Whitney test) when compared to the respective M1 cluster properties expressed without HA (Figure 11). The mean density of the M1 cluster expressed without HA was slightly greater compared to the mean density of the M1 cluster expressed with HA, but was not observed to be significantly different with the one-tailed Mann–Whitney test. Overall, these results show the role of HA in enhancing the M1 clustering (area, perimeter, and number of molecules, but not density) at the PM.

## 4. Discussion

The matrix protein (M1) is the most abundant viral protein in IAV, and has lipid-binding domains [12,63,64]. M1 in the cytoplasm binds to the PM primarily due to electrostatic interactions [13,65], suggesting it can interact with various anionic lipids [9], even in the absence of any other viral proteins, although interaction with the membrane is enhanced by NA and acylated HA in a curvature-dependent manner [18,19]. M1 contains a positively charged face proposed to interact with the cytoplasmic leaflet of the PM and/or vRNP [20,23]. M1 is significantly more likely to be adsorbed onto surfaces containing negatively charged phospholipids than onto surfaces with a neutral charge [24]. PIP2, which is the most abundant PM polyphosphoinositide [66,67], presumably has a net charge of −3 at a neutral pH [68], suggesting the potential for electrostatic interaction with positive residues in M1. Many cellular proteins, for example, are known to interact with PIP2 through polybasic residues. [69]

CPC consists of a positively charged head group with a long hydrophobic tail. At concentrations in the range of millimolar, CPC disrupts membrane integrity and exhibits antibacterial and antiviral properties [49,50,51]. We previously showed that CPC is non-cytotoxic at concentrations in the range of 5–10 μM, and displaces some PIP2-binding proteins from the PM, presumably by disrupting electrostatic interactions [42]. We also found that HA clustering and HA-PIP2 co-clustering were disrupted by the same concentrations of CPC, and outcomes of IAV infection in vivo were improved by treatment with CPC [42]. Thus, we wanted to test if M1 is also associated with PIP2 and if CPC could disrupt this association or disrupt the association between HA and M1. To test these questions, we used the super-resolution method of FPALM.

### 4.1. CPC Disrupts M1 and PIP2 Colocalization, Clustering, and Co-Clustering

To test the association of IAV M1 and PIP2, we co-transfected cells with M1-PAmKate and Dendra2-PH (PLC-δ), which are established markers of PIP2 [70,71,72,73]. The PH domain of PLC-δ binds to PIP2 with relatively high specificity, and therefore can be used as a marker of the spatial distribution of PIP2; the PH domain from PLC-δ has been extensively used in previous studies [72,74,75]. Dendra2-PH has been used previously to label PIP2 [6,42,59]. PAmKate is a known super-resolution probe derived from mKate [76], and is spectrally well separated from Dendra2, which makes it compatible with two-color super-resolution microscopy with acceptably low bleed-through between the probes [6,53]. Fluorophores such as YFP and mCardinal, whose sizes are similar to PAmKate, were successfully used to label M1 [17,22]. However, since M1 interacts with the lipid head groups electrostatically through the positive charges in the N-terminal fragment [77,78], PAmKate was cloned to be expressed at the C-terminal of M1. Previous studies showed that some of the functions of M1 were impaired when a fluorescent protein was cloned for expression at the N-terminal of M1 [79], suggesting that the C-terminal location of M1 could avoid some of those problems. As expected, the results showed some plasma membrane association and clustering of M1 in the absence of other viral components, with clustering over a wide range of length scales (30–500+ nm; Figure 1) and a mean cluster area equivalent to a circular cluster of radius ~200 nm (Figure 4), consistent with M1 clustering on length scales of 20–200+ nm reported previously [21].

PIP2 is a minor PM lipid component and yet is responsible for a large number of cellular functions [28,68,80]. PIP2 has previously been shown to cluster at the PM [6,33,34]. PIP2-binding domains in proteins typically interact with PIP2 through electrostatic interactions and hydrogen bonds and can bind to negatively charged PIP2 and PIP3 [69]. Our results show CPC significantly disrupts the clustering of the PH domain in the PM (Figure 2), consistent with our previous result demonstrating the effect of CPC on PIP2-binding proteins [42]. Our reported PH domain cluster area (control group) is in good agreement with our previously reported PH domain cluster area [42].

In addition, regions of colocalization between M1 and PH domain were observed (Figure 1) and CPC treatment reduced that colocalization significantly, as quantified by MCC (Figure 2). This result suggests that CPC, whose head group is positively charged, disrupts the association of PIP2 and M1, possibly by interfering with the electrostatic interactions with which M1 may bind to PIP2.

M1 multimerizes on binding to a lipid bilayer [17]. This higher-order clustering of M1 may have an important role in the viral life cycle, as M1 plays a critical role in virion assembly, and this clustering is thought to induce membrane curvature [25]. Additionally, it has been previously shown that M1 can produce VLPs on its own [14], but there is very little quantitative knowledge about the lipid-dependence of the nanoscale clustering of M1. Hence, we studied and quantified the cluster properties of M1 at the PM and its dependence on PIP2 and CPC. While three of the cluster properties (area, perimeter, and the number of molecules forming a cluster) were disrupted by CPC, the density remained unaffected (Figure 4). Since the CPC is expected to inhibit the binding of M1 to anionic lipids, it is understandable that M1 clusters are reduced in size. However, not all M1 molecules are disrupted from binding to the membrane. This could be due to the ability of M1 to self-assemble into clusters once it binds to other lipid components of the membrane [17,81], or due to some remaining inner leaflet anionic lipids, including phosphatidylserine, which may or may not be neutralized by the CPC. Note that the distance threshold (d_max_) for clustering puts a lower limit on the density D of a cluster which can be identified by our methods, i.e., D_min_ = 1/(d_max_^2^). In general, it is not possible to observe clusters with D < D_min_, because at such lower densities, the molecules will be farther apart than d_max_ and clusters will not be detected. Accordingly, the area of the cluster may decrease to include only those regions with a density larger than the minimum. If the cluster has regions with different densities, the higher-density regions may remain detected by the algorithm, while the others may be lost. However, if the cluster is a uniform density, then it will either be detected if its density is above the minimum detectable density, or it will be missed altogether if its density is lower than the minimum.

In addition to cell-wide “global” cluster quantification, we were also interested in whether CPC disrupted the co-clustering of PIP2 and M1 locally. To determine the local effect of CPC on PIP2 and M1, the co-clustering of PIP2 and M1 was quantified using the Mean Pixel Sum method. PIP2 molecules (measured by PH domain clustering) co-clustering with M1 (Figure 5A) and M1 molecules co-clustering with PIP2 (measured by PH domain clustering) decreased in number significantly with CPC treatment (Figure 5B). Building on Raut et al.’s 2022 study [42] which shows that CPC reduces the membrane association of PIP2-binding proteins, these results show that CPC also disrupts M1 clustering, and show that M1 clusters in the PM are mediated by the PM distribution of PIP2, further suggesting a possible interaction between PIP2 and M1.

### 4.2. Influenza A M1 Enhances PIP2 Clustering

The finding that CPC disrupts the co-clustering of M1 and PIP2 (Figure 5) suggests a possible interaction between PIP2 and M1. We decided to further explore this possibility by quantifying cluster properties of PIP2 expressed with and without M1. We observed that in the presence of M1, PIP2 cluster area and perimeter significantly increased (Figure 6), and more PIP2 molecules were incorporated into the PIP2 cluster, while no change in the density was observed (Figure 6). This enhancement in PIP2 clusters by M1 further suggests a possible interaction between PIP2 and M1. While we cannot yet distinguish a direct or indirect mechanism of interaction, further details of interaction could be investigated in the future using molecular dynamics and experimental methods with access to even shorter length scales. Other phosphoinositides present at the PM, or elsewhere along the route taken by M1 from synthesis to the PM, could also interact with M1 and would be worth investigating.

### 4.3. CPC Disrupts the Assembly of HA and M1

It has been previously suggested that IAV M1 interacts with the CTD of HA, which might be crucial for the viral life cycle [19,26,27]. The quantitative distributions of the viral proteins NA with HA and M2 with HA have previously been studied, but the effect of PIP2 on M1 clustering and HA-M1 co-clustering has not been explored [82]. Our previous studies have shown that HA clustering is modulated by PIP2 clustering, and that the co-clustering of HA and PIP2 is reduced by CPC [42]. With these findings in mind, we decided to examine the effect of CPC on HA-M1 co-clustering mediated by PIP2. Super-resolution images, using TIRF excitation to limit the observation of cellular regions close to the PM, showed reduced regions of co-clustering of HA and M1 with CPC treatment (Figure 7). Next, we quantified the colocalization of HA and M1 using the mean pixel sum and MCC (Figure 8) as a function of CPC treatment. We observed that CPC significantly reduced both the Mean Pixel Sum and the MCC. Thus, CPC, which reduces the association of PIP2 with PIP2-binding proteins in the PM [42], also reduces the PM association of M1 with HA.

In addition to the HA clusters, M1 cluster properties were quantified as a function of CPC treatment (Figure 9). While the cluster properties such as area, perimeter, and the number of M1 molecules per cluster were significantly disrupted by CPC, the density of M1 clusters remained fairly similar, and no significant difference was observed. Given that the multimerization of M1 is important for viral assembly, these findings suggest that the alteration of phosphoinositide levels in the PM could be further investigated as a strategy to inhibit IAV infection.

To further test the mechanism by which HA could mediate the PM distribution of M1, we quantified the effect of HA expression on the number of M1 localizations at the PM. We did not observe any significant difference in the number of PM localizations of M1 in cells’ ±HA expression (Figure 10), which suggests a limited role of HA in the PM association of M1, at least at our level of detection. However, a significant difference was observed in the M1 cluster properties when M1 was expressed with and without HA: in the presence of HA, M1 cluster area, perimeter, and the number of M1 molecules forming a cluster all increased significantly (Figure 11). The separation between HAs within viral-like particles was observed previously to depend on the presence of M1 [18], but the changes in spacing (~6%) are likely too small for our methods to resolve; we did not observe a significant change in M1 density ±HA. Leser and Lamb [21] studied the distribution of HA and M1 in both IAV-infected and transfected cells and clearly demonstrated the co-clustering of HA and M1, but the dependence of HA and M1 clustering on phosphoinositides was not reported. In addition, our observation of the increased area and number of molecules of M1 per cluster in the presence of HA is distinct (Figure 11), showing that M1 cluster size, but not density, is enhanced strongly by HA, indicating a possible PIP2-dependent role of HA in the incorporation of M1 into assembling virions. Because M1 can induce lipid deformation upon multimerization [25], interactions between PIP2, HA, and M1 could play a role in the degree or rate of budding. These interactions could also play a role in the density of HA clusters which form on the surface of nascent viral particles. Since high HA cluster density is correlated with an increased probability of HA-dependent membrane fusion [83], viral particles which contain high-density HA clusters may be more infectious once released [84]. Thus, multiple aspects of the IAV life cycle could be affected by PM phosphoinositide levels, suggesting phosphoinositides as worth further investigation in the search for novel antiviral strategies.

## 5. Conclusions

M1 is the most abundant IAV protein, can interact directly with lipid bilayers, and can form VLPs in the absence of other viral proteins [14]. The cytoplasmic tails of HA and NA have also been suspected in the interaction of M1 with the viral surface glycoproteins and host cell membranes, although the mechanism of interaction remains controversial [18,20,85,86]. Detergent resistance studies show contradicting results in terms of the membrane association of M1 mediated by the viral surface glycoproteins [62,77].

The interaction of M1 with lipid bilayers is considered to be primarily electrostatic [10,11,12,13,20] and M1 can bind lipid bilayers through multiple residues [9]. M1 is known to interact with PS in cells and cell models [22], but M1 interaction with other lipids has not been extensively studied. If interactions between M1 and lipid bilayers are primarily electrostatic, it is plausible that M1 could interact with lipids that are more negatively charged than PS. We tested this hypothesis and found M1 colocalized with the PLCδ-PH domain, an established marker of PIP2. The finding that PIP2 clustering was significantly enhanced in the presence of M1 is consistent with the response of PIP2 clustering to other positively charged membrane-associated molecules [42] and ions [87] and the positively charged face of M1 [23], supporting the notion that M1 interacts with PIP2 electrostatically at the PM.

The quaternary ammonium compound CPC, found previously to disrupt PIP2 clustering and reduce the PM association of PIP2-binding proteins at low micromolar concentrations [42], significantly disrupted the colocalization, clustering, and co-clustering of HA and M1, as well as the colocalization, clustering, and co-clustering of M1 and PIP2. CPC also improved the outcomes of IAV infection in vivo [42].

The implicated role of PIP2 in HA-M1 interaction and M1 association with the PM is novel, suggesting that it may be fruitful to explore strategies which modulate host cell PIP2 or other phosphoinositides to prevent or inhibit IAV infection. 

## Data Availability

Raw image files supporting this study will be provided upon reasonable request to the corresponding author.
